# Two-dimensional reduced graphene oxide as high-efficiency hole injection layer for quantum dot light-emitting diodes

**DOI:** 10.1039/d6ra00068a

**Published:** 2026-03-12

**Authors:** Suwen Yang, Ning Wang, Yufeng Hu, Zhidong Lou, Yanbing Hou, Feng Teng, Yu Zhang

**Affiliations:** a Key Laboratory of Luminescence and Optical Information, Ministry of Education, School of Physical Science and Engineering, Beijing Jiaotong University Beijing 100044 China fteng@bjtu.edu.cn zhyu@bjtu.edu.cn

## Abstract

Carrier injection imbalance severely limits the performance of quantum dot light-emitting diodes (QLEDs), emphasizing the demand for advanced transport layer materials. Herein, a high-performance reduced graphene oxide (rGO) hole injection layer (HIL) is prepared by thermally treating graphene oxide (GO) at 160 °C for 30 min, which boosts current density by two orders of magnitude, and tunes work function to 5.04 eV, thus lowering hole injection barriers. rGO-based QLEDs exhibit excellent optoelectronic performance, featuring a 2.0 V turn-on voltage and a maximum luminance of 120 000 cd m^−2^. Their peak external quantum efficiency (EQE) and current efficiency are enhanced from 8.07% and 8.99 cd A^−1^ (for same-batch GO-based devices) to 11.51% and 12.65 cd A^−1^. Further optimization elevates their peak EQE and current efficiency (CE) to 13.31% and 14.93 cd A^−1^, respectively. Performance gains stem from enhanced rGO conductivity, with rGO-based devices boasting superior thermal stability and low-temperature operability. This study verifies thermally reduced rGO as an ideal high-performance HIL, offering a new possibility for QLED optimization.

## Abstract 2

rGO-based QLEDs achieve excellent performance (2.0 V turn-on voltage, 120 000 cd m^−2^ maximum luminance, 11.74%EQE), on par with same-batch PEDOT:PSS-based devices.

## Graphical abstract

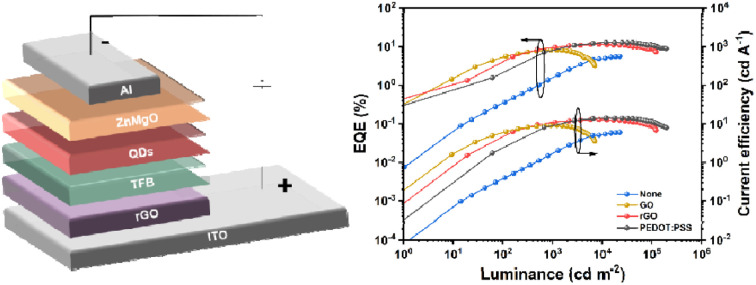

## 1. Introduction

Quantum dot light-emitting diodes (QLEDs) exhibit tremendous application potential in next-generation display and lighting technologies, owing to their prominent advantages such as high color purity, precise tunability of emission color *via* size adjustment, and solution processability.^1,2^ The efficient luminescence of QLEDs relies on the balanced injection and recombination of electrons and holes within the QD emissive layer.^3^ However, commonly used electron transport materials (*e.g.*, ZnO, TiO_2_) possess excellent electron injection capability.^4,5^ In contrast, the hole injection side typically faces a high barrier that impairs hole injection efficiency, resulting in prevalent carrier injection imbalance in QLED devices. This imbalance further induces critical challenges such as efficiency roll-off, increased turn-on voltage, and degraded device stability.^6,7^ Thus, developing high-performance hole injection layers (HILs) to optimize carrier balance is pivotal for enhancing the overall performance of QLEDs.^8^ Currently, poly(3,4-ethylenedioxythiophene):poly(styrenesulfonate) (PEDOT:PSS) is the most widely used solution-processable HIL material.^9^ Nevertheless, the strong acidity of PEDOT:PSS can corrode the indium tin oxide (ITO) anode, and its hygroscopicity tends to introduce moisture and oxygen, accelerating device aging. These inherent drawbacks severely limit the long-term operational stability of QLEDs.^10,11^ To explore alternatives, researchers have investigated various materials such as transition metal oxides (*e.g.*, MoO_3_,^12^ V_2_O_5_,^13^ WO_3_,^14^ and NiO_*x*_^15^), but the comprehensive device performance or solution processability still has limitations.^16^

Graphene oxide (GO), an emerging two-dimensional carbon material, has garnered widespread attention in the interface engineering of optoelectronic devices owing to its superior properties—including excellent solution processability, high optical transmittance, and matched energy level structure.^17,18^ However, GO is inherently an electrical insulator, and its ultra-low conductivity severely restricts hole transport capability. Consequently, QLEDs based on pure GO hole injection layers (GO-HILs) typically exhibit far inferior performance to those using PEDOT:PSS.^19^ Song *et al.* explicitly noted that the hole current of GO-HILs is one order of magnitude lower than that of their reduced derivative (rGO), with a notable performance gap.^20^ To address GO's poor conductivity bottleneck, researchers have explored various optimization strategies. Chen *et al.* doped GO into PEDOT:PSS to fabricate a composite hole injection layer (PEDOT-GO).^21^ They observed that GO incorporation modulates the composite's work function, shifting its valence band downward to better align with QD's energy level. This reduces the hole injection barrier and enhances device performance.^22^ Nevertheless, this approach fails to fully eliminate reliance on PEDOT:PSS, leaving its inherent acidity and instability issues unresolved.^23^ Reduced graphene oxide (rGO) significantly enhances material conductivity by partially restoring graphene's sp^2^ conjugated structure, while its work function can be tuned *via* the degree of reduction.^24,25^ Song *et al.* prepared rGO through ultraviolet (UV) reduction of GO, achieving conductivity over one order of magnitude higher than that of GO. Then QLEDs based on rGO-HILs exhibited more than a twofold improvement in both current efficiency (CE) and external quantum efficiency (EQE) compared to GO-based devices.^20^ The single-hole device tests and energy level analysis identified enhanced conductivity as the dominant driver of performance improvement.^26^ Similarly, Yan *et al.* demonstrated that ultraviolet ozone (UVO)-treated graphene oxide (GO)—deemed a mild reduction process—yields quantum dot light-emitting diode (QLED) devices with substantially enhanced luminance and current efficiency when employed as the hole injection layer (HIL), compared to poly(3,4-ethylenedioxythiophene):poly(styrenesulfonate) (PEDOT:PSS)-based counterparts.^27–29^ These studies strongly validate the feasibility of rGO as a PEDOT:PSS replacement. However, most existing research employs UV reduction or focuses on GO/PEDOT:PSS composites, with overall device performance remaining relatively low. In contrast, thermal reduction is a straightforward process that requires no additional chemical reducing agents and is easily integrable into device fabrication workflows. Yet, QLEDs incorporating standalone rGO-HILs prepared *via* thermal reduction warrant further investigation.^30^ In particular, systematically comparing the performance of thermally reduced rGO with that of GO and PEDOT:PSS, and elucidating how thermal reduction synergistically optimizes rGO's conductivity and interfacial energy level alignment, is critical for advancing the practical application of rGO as a high-performance hole injection layer in QLEDs.^31,32^

Accordingly, this study proposes a one-step thermal reduction method to directly fabricate a rGO-HIL on indium tin oxide (ITO) substrates, ultimately constructing Cd-based QLEDs integrated with the rGO-HIL. We systematically investigated the effects of annealing temperature and time on the degree of graphene oxide (GO) reduction and consequently on QLED performance, identifying the optimal annealing conditions. QLEDs with this rGO-HIL exhibit excellent optoelectronic performance: a low turn-on voltage of 2.0 V, a maximum luminance of 120 000 cd m^−2^, and enhanced EQE and current efficiency (from 8.07% and 8.99 cd A^−1^ to 11.51% and 12.65 cd A^−1^, respectively) compared to GO-based devices. The performance is comparable to PEDOT:PSS-based devices while far superior to GO-based counterparts. Further analysis confirms that the significant enhancement in rGO conductivity is the primary driver of the improved device optoelectronic performance. This work provides a novel solution and experimental foundation for exploring new materials as hole injection layers (HILs) in high-performance, high-stability quantum dot light-emitting diodes (QLEDs).

## 2. Experimental

### 2.1 Fabrication of QLED devices

Patterned indium tin oxide (ITO) glass substrates were sequentially ultrasonicated in ITO cleaning solution, deionized water, and isopropanol for 15 minutes each, followed by drying with high-purity nitrogen gas. Subsequently, the substrates were treated with plasma for 3 minutes to thoroughly remove organic contaminants and enhance the work function and hydrophilicity of the ITO surface.^33–39^ Tree types of hole injection layers (HILs) were fabricated for comparative studies, with the detailed processes as follows. For the PEDOT:PSS HIL, the aqueous dispersion of poly(3,4-ethylenedioxythiophene):poly(styrenesulfonate) (PEDOT:PSS) was spin-coated onto the plasma-treated ITO substrates at 5000 rpm for 30 seconds, followed by thermal annealing in ambient air at 150 °C for 30 min. For the rGO HIL, the aqueous dispersion of graphene oxide (GO, 5 mg mL^−1^) was spin-coated onto the ITO substrates at 3000 rpm for 30 seconds, then annealed at 160 °C for 30 minutes to obtain the rGO layer. After annealing, the substrates were transferred into a nitrogen (N_2_)-filled glove box, where TFB (8 mg mL^−1^) was spin-coated at 4000 rpm and annealed at 150 °C for 30 minutes. The toluene dispersion of red-emitting CdSe/ZnS core–shell quantum dots (QDs, concentration: 15 mg mL^−1^) was spin-coated onto the TFB layer at 3000 rpm to form a uniform and dense emissive layer. The laboratory-synthesized ethanol dispersion of zinc magnesium oxide nanoparticles (ZnMgO NPs, concentration: 30 mg mL^−1^) was spin-coated onto the QD layer at 2000 rpm for 30 seconds, followed by annealing at 60 °C for 30 minutes to form the electron transport layer. The substrates with multi-layer thin films were transferred into an ultra-high vacuum thermal evaporation system (chamber pressure < 5 × 10^−4^ Pa), where an aluminum (Al) metal electrode (∼100 nm thick) was deposited. The effective light-emitting area of the devices was defined as 4 mm^2^ using a precision metal mask. Finally, the devices were encapsulated with ultraviolet (UV)-curable glue.

### 2.2 Sample characterization

An X-ray Photoelectron Spectrometer (XPS, Escalab 250Xi, Thermo Scientific, USA) was used to analyze the surface chemical states of the materials. A Raman spectrometer (alpha300, WITec, Germany) was utilized to obtain Raman spectra.A field-emission Scanning Electron Microscope (SEM, SU8020, Hitachi, Japan) was employed to observe the sample morphology at an operating voltage of 3.0 kV. An Atomic Force Microscope (AFM, Dimension Icon, Bruker, USA) was used to analyze the surface roughness. Fluorescence spectra were determined with a HORIBA Fluorolog-3 fluorescence spectrometer. Ultraviolet-visible (UV-vis) absorption spectra were measured using a SHIMADZU UV3600iplus UV-vis-NIR spectrophotometer. An electrical and optical performance testing system (LQ-50X, Enlitech, Taiwan, China) was adopted to characterize the optoelectronic properties of the QLED devices.

## 3. Results and discussion

**Fig. 1:**
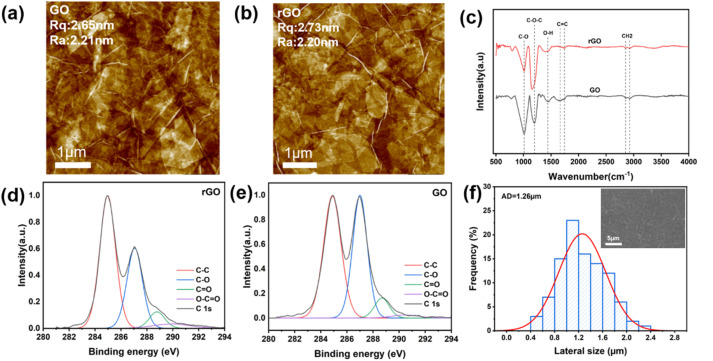
Structural and morphological characterizations of GO and rGO (rGO annealed at 160 °C for 30 min). (a) AFM image of GO film. (b) AFM image of rGO film. (c) FTIR spectra of GO and rGO. (d) High-resolution C 1s XPS spectrum with peak deconvolution of rGO. (e) High-resolution C 1s XPS spectrum with peak deconvolution of GO. (f) Lateral size distribution of rGO nanosheets; inset: corresponding SEM image of rGO film.

Graphene oxide (GO) and reduced graphene oxide (rGO) are typical graphene derivatives featuring facile accessibility and low cost. Unlike zero-bandgap graphene, GO possesses a wide bandgap, and its carrier injection capability diminishes with increasing thickness.^20^ A suite of characterizations was performed to investigate the chemical composition, functional group variations, morphology, and size characteristics of the GO and rGO employed in this work. The rGO utilized here was readily obtained *via* direct annealing of GO film at the optimal condition of 160 °C for 30 min. Atomic force microscopy (AFM) was employed to characterize the surface morphology and roughness of GO and rGO, as illustrated in Fig. 1(a) and (b). The root-mean-square roughness (*R*_*q*_) and arithmetic mean roughness (*R*_*a*_) values of GO are 2.65 nm and 2.21 nm, respectively, *versus* 2.73 nm and 2.20 nm for rGO. These comparable roughness parameters indicate that the thermal reduction process did not notably alter the morphology or surface roughness of the GO film.^40–42^ Moderate nanoscale roughness of the rGO surface enhances the effective contact area with the emissive layer to promote hole injection from the HIL to the QD layer, while also supporting uniform, dense deposition of subsequent functional layers.^34^Fig. 1(c) presents the normalized Fourier transform infrared (FTIR) spectra of GO and rGO (Table S1). For the GO spectrum (black line), characteristic peaks correspond to: C–O stretching vibration at 1005 cm^−1^, epoxy C–O–C stretching vibration at 1192 cm^−1^, O–H bending vibration at 1440 cm^−1^, C=C skeletal vibration at 1634 cm^−1^, and carboxyl C=O stretching vibration at 1737 cm^−1^. These peaks collectively confirm the presence of abundant oxygen-containing functional groups in GO.^40–46^ In contrast, the intensities of oxygen-containing functional group peaks (*e.g.*, C–O) are significantly attenuated in the rGO spectrum (red line). This verifies that the reduction process effectively eliminates oxygen-containing functional groups and restores the conjugated structure of graphene,^24^ yielding rGO. The restoration of this conjugated structure is critical for rGO to achieve excellent conductivity, enabling it to act as a high-performance carrier transport layer.^25,31^ Meanwhile, X-ray photoelectron spectroscopy (XPS) was utilized to comparatively analyze the chemical composition of GO before and after annealing (Fig. S1). Fig. 1(d) presents the high-resolution C 1s XPS spectrum of GO, which can be deconvoluted into four characteristic peaks: C–C (∼284.6 eV), C–O (∼286.2 eV), C=O (∼287.8 eV), and O–C=O (∼289.0 eV).^44^ The intense signals of oxygen-containing functional groups (C–O, C=O, O–C=O) confirm the presence of abundant hydroxyl, epoxy, and carboxyl groups on the GO surface, thereby establishing the structural basis for its excellent hydrophilicity and reactivity. Fig. 1(e) shows the high-resolution C 1s XPS spectrum of rGO. Relative to GO, the C–O peak intensity is significantly diminished, while the C–C peak proportion is substantially enhanced. This demonstrates that thermal reduction effectively eliminates surface oxygen-containing functional groups and restores the sp^2^ carbon skeleton, which is consistent with FTIR findings—collectively confirming the successful conversion of GO to rGO. XPS quantitative analysis (Table 1) reveals that the total oxygen content decreases from 50% (GO) to 44% (rGO). Notably, the residual trace oxygen-containing functional groups in rGO impart moderate hydrophilicity and surface active sites, facilitating improved interfacial contact with adjacent functional layers in the device.^41^ Besides, the UV-visible (UV-vis) transmittance spectra of GO and rGO also has been conducted, showing no obvious difference, as shown in Fig. S2. Fig. 1(f) presents the statistical distribution of the lateral size of rGO, with the inset showing the corresponding scanning electron microscopy (SEM) image (Fig. S3). Statistical analysis of 100 rGO nanosheets indicates an average lateral size of ∼1.26 µm, with sizes predominantly distributed in the range of 0.8–1.6 µm. This relatively large lateral size facilitates the formation of a continuous, intact conductive network during rGO film fabrication, thereby reducing interfacial barriers and scattering effects in hole transport and boosting hole injection and transport efficiency.^19,41^ In brief, these results confirm that annealing treatment effectively eliminates oxygen-containing functional groups from GO, restores the sp^2^ conjugated carbon structure, and successfully yields rGO—laying a robust material foundation for the subsequent fabrication of QLED devices.

**Table 1:** The XPS data of GO and rGO samples

XPS spectral area	C–C/C=C	C–O	C=O	O–C=O	
Binding energy [eV]	284.9	286.8	288.7	290.5	Total oxygen concentration
GO	49.9	41.5	6.8	1.9	50%
rGO	55.7	33.0	6.8	4.4	44%

**Fig. 2:**
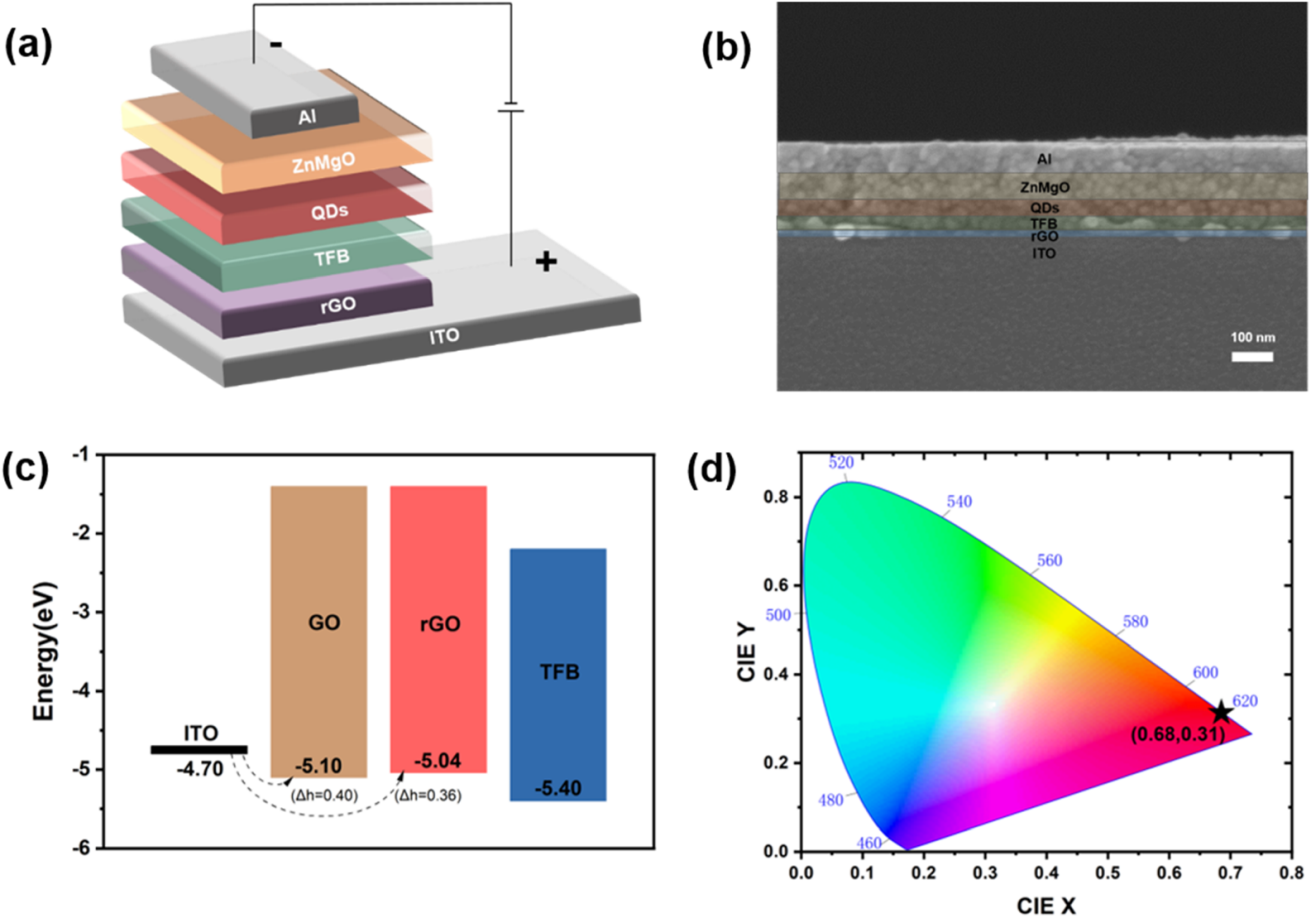
Structural and optoelectronic characterizations of QLEDs. (a) Device structure diagram. (b) Cross-sectional SEM image of the device. (c) Energy level diagram of the device. (d) CIE chromaticity coordinates.

To systematically investigate the performance of rGO as a HIL in QLEDs, devices with the structure glass/ITO/rGO/TFB/QDs/ZnMgO/Al were fabricated *via* an all-solution process, as depicted in Fig. 2(a). Specifically, rGO acts as the HIL positioned between the ITO anode and the hole transport layer (TFB), fulfilling the critical roles of hole injection and transport. Fig. 2(b) shows the cross-sectional scanning electron microscopy (SEM) image of a Cd-based QLED, with well-defined interfaces among ITO/rGO/TFB/QDs/ZnMgO/Al and a distinct Al layer. From the cross-sectional image, the thicknesses of each functional layer are quantitatively measured as follows: ∼10 nm for the rGO HIL, ∼30 nm for the TFB layer, ∼35 nm for the QD emissive layer, ∼65 nm for the ZnMgO electron transport layer (ETL), and ∼80 nm for the Al electrode. The uniform and compact layered configuration, together with the well-controlled layer thicknesses, is very important for efficient carrier transport and recombination, thereby effectively mitigating carrier trapping or leakage current issues induced by interfacial defects.^6,38^Fig. 2(c) illustrates the energy level alignment of the fabricated device. The work functions of GO and rGO were derived from ultraviolet photoelectron spectroscopy (UPS), as detailed in Fig. S4. ITO exhibits a work function of −4.70 eV, whereas GO has a work function of −5.10 eV—resulting in an energy level offset (Δ*h*) of 0.40 eV at the GO/ITO interface. In contrast, rGO features a work function of −5.04 eV, which reduces the ITO/rGO energy level offset to 0.36 eV. This reduced energy level offset signifies a substantial lowering of the hole injection barrier between ITO and rGO, which is conducive to facilitating efficient hole injection from the anode to the HIL. Furthermore, the energy level offset between rGO and TFB is less than 0.4 eV, a value that favors the formation of ohmic contact and enables favorable energy level matching for the subsequent efficient transport of holes. This optimized energy level alignment is one of the crucial prerequisites for rGO to enhance the hole injection efficiency of the QLED device.^26^ The CIE color coordinates serve as a standard system for evaluating color performance. Fig. 2(d) shows the CIE color coordinate diagram of the QLED device based on the rGO-HIL, with color coordinates of (0.68, 0.31) corresponding to red light emission around 620 nm. This indicates that replacing the commonly used PEDOT:PSS with rGO as the HIL does not affect the emission color. Instead, the device retains deep red emission with high color purity and saturation, rendering it well-suited for high-color-gamut display applications. Fig. S5 presents the electroluminescence (EL) spectra of the device under different voltages. As the driving voltage increases, the emission intensity is significantly enhanced, while the peak wavelength and full width at half maximum (FWHM) of the EL spectrum remain basically unchanged. This demonstrates that the QLED device has stable emission color and high color purity under different voltages, which is a crucial performance advantage for display devices.

**Fig. 3:**
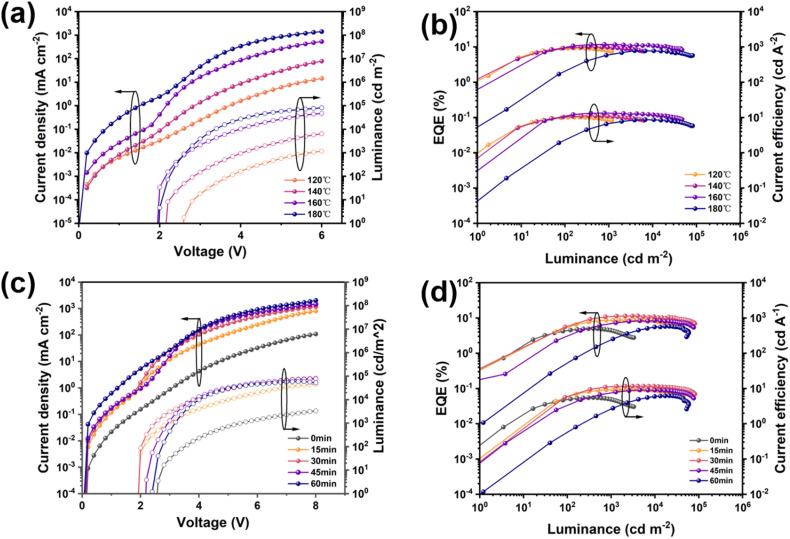
Performance comparison of QLEDs with rGO-HILs. (a) Current density and luminance *versus* voltage (*J*–*V*–*L*) curves at different temperatures. (b) External quantum efficiency (EQE) and Current efficiency (CE) *versus* luminance curves at different temperatures. (c) *J*–*V*–*L* curves at different annealing times. (d) EQE and CE *versus* luminance curves at different annealing times.

To this end, thermal annealed rGO was employed as an efficient HIL in QLEDs. To further clarify how rGO with different reduction degrees affects device performance, two key parameters—annealing temperature and annealing time—were systematically modulated to prepare rGO samples under distinct conditions. Subsequently, the corresponding QLED device performance were evaluated when these tailored rGO samples were integrated as the HIL. Thermal annealing temperature is a key parameter governing the reduction degree and electrical properties of rGO. Initially, spin-coated GO films were subjected to thermal annealing at different temperatures with a constant annealing duration maintained. Fig. 3(a) depicts the current density–voltage–luminance (*J*–*V*–*L*) characteristic curves of QLED devices with rGO thermally annealed at different temperatures (120 °C, 140 °C, 160 °C, and 180 °C). As the annealing temperature increased from 120 °C to 160 °C, the turn-on voltage of the devices gradually decrease from 2.6 V to 2.0 V. This result clearly demonstrates that elevating the annealing temperature effectively enhances the reduction degree of rGO, optimizes the energy level alignment at the electrode/functional layer interface, and thereby minimizes the hole injection barrier (Fig. S6). Concomitantly, the device current density exhibited an order-of-magnitude increase as annealing temperature increased, with the device incorporating rGO annealed at 160 °C achieving the highest current density and luminance values (see Table S2 for detailed device performance parameters). This phenomenon underscores that moderate thermal annealing can effectively improve the crystallinity and electrical conductivity of rGO. However, further elevating the annealing temperature to 180 °C led to noticeable device performance deterioration, which can be ascribed to excessive agglomeration of rGO nanosheets or thermal decomposition of functional layer material. Fig. 3(b) illustrates the corresponding external quantum efficiency (EQE)-luminance and current efficiency (CE)-luminance characteristics of devices with rGO-HILs annealed at different temperatures. Among these, the QLED integrated with the 160 °C-annealed rGO-HIL achieves the highest peak EQE (11.74%) and the mildest efficiency roll-off behavior, which attests to the fact that this annealing temperature enables the optimal balance of carrier injection and recombination dynamics.^7,20^ Subsequently, we fixed the annealing temperature and investigated the influence of varying annealing durations (0 min, 15 min, 30 min, 45 min, and 60 min) on the performance of rGO-based QLEDs. Fig. 3(c) and (d) illustrate the optoelectronic characteristics of QLED devices with rGO as the HIL annealed for different durations. As the annealing duration increased, the device current density and luminance first increased significantly and then plateaued. This indicates that the reduction rate is higher in the early stage of annealing, leading to a more pronounced reduction effect, and the device with rGO reduced for 30 min annealing exhibited the optimal performance. When the annealing duration is insufficient, rGO retains a high content of residual oxygen-containing functional groups, which results in inferior electrical conductivity and an imbalance between hole and electron injection.^24,29^ To gain insight into the reduction degree of rGO, Raman spectroscopy characterization was performed on rGO samples with different annealing durations, as shown in Fig. S7(a) and Table S3. The D band arises from defects, the peak area ratio *A*_D_/*A*_G_ of the D and G bands is used to evaluate the defect concentration in rGO. Meanwhile, the peak areas of the D and G bands are dependent on the reduction degree of graphene oxide. As illustrated in Fig. S7(b), the *A*_D_/*A*_G_ area ratio increases with prolonged annealing duration, indicating the formation of additional sp^2^-hybridized carbon structures during the reduction process. While this structural evolution is conducive to enhancing the electrical conductivity of rGO, the density of structural defects also increases concomitantly. Consequently, when the annealing duration is extended to 45 min and 60 min, the device performance exhibits a gradual degradation, with the EQE decreasing from 11.46% to 9.04% and further declining to 6.22%. Thus, an annealing duration of 30 min achieves the optimal balance between the electrical conductivity and structural integrity of rGO, leading to the highest hole injection efficiency for the QLED devices (Table S4).

**Fig. 4:**
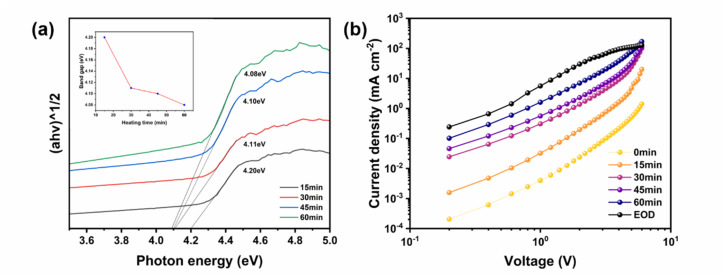
The mechanism of rGO-HIL in QLEDs. (a) Tauc plots of ZnO between (*αhν*)^2^*versus* photon energy. (inset: bandgap evolution), (b) charge transport in single-carrier devices.

To further elucidate the effect of rGO with varied annealing durations on electron injection dynamics, the band structures and electronic properties of the rGO samples were systematically investigated. Fig. 4(a) illustrates the Tauc plots derived from the absorption spectra of rGO samples annealed for different times (Fig. S8), with the inset showing the variation of band gap with annealing duration. As the annealing duration increases, the band gap of rGO gradually decreases (from 4.30 eV to ∼4.10 eV), which is ascribed to the expansion of sp^2^-conjugated domains and the increased continuity of energy levels following the elimination of oxygen-containing functional groups.^25,31^ This reduced band gap not only optimizes the energy level alignment between rGO and ITO/TFB, thereby minimizing the hole injection barrier, but also enhances the electrical conductivity of rGO to enable more efficient hole injection. Consequently, based on the systematic investigation of annealing temperature and annealing time, the optimal conditions for converting GO to rGO *via* thermal annealing in our system are defined as: an annealing temperature of 160 °C and an annealing duration of 30 min. The QLED devices integrated with rGO-HIL synthesized under these optimal conditions exhibit the superior overall performance. To further elucidate the regulatory effect of annealing duration on hole injection, we fabricated electron-only devices (EODs) and hole-only devices (HODs) with the following structures: ITO/ZnMgO/TFB/QD/ZnMgO/Al and ITO/rGO(n)/TFB/QD/MoO_3_/Al, where “n” denotes the annealing duration, set to 0 min, 15 min, 30 min, 45 min and 60 min, respectively. Fig. 4(a) presents the current–voltage (*I*–*V*) characteristic curves of the EODs and HODs. It is observed that the hole current increases by nearly 1000-fold with prolonged annealing duration, demonstrating that thermally reduced rGO can effectively mitigate the imbalance between hole and electron injection.

**Fig. 5:**
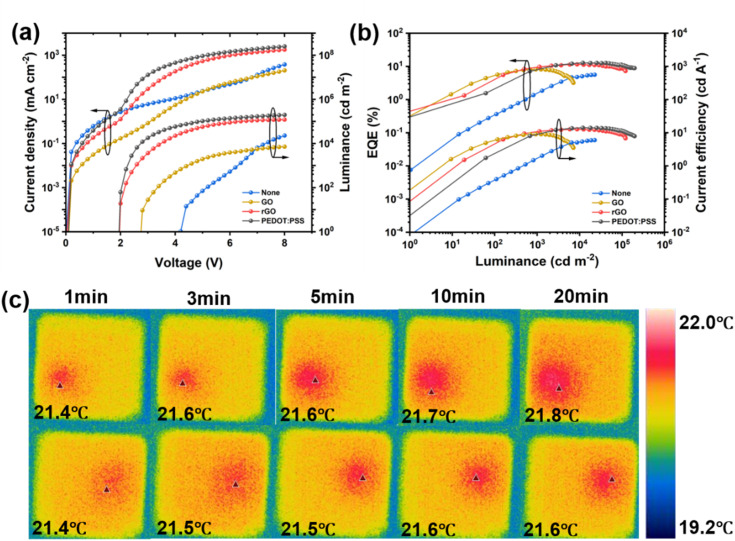
Performance comparison of QLEDs with different HILs. (a) *J*–*V*–*L* characteristics of QLEDs with different HILs; (b) EQE-Luminance and CE-Luminance characteristics of QLEDs with different HILs; (c) thermal stability test.

Finally, we systematically compared the performance of QLED devices integrated with different hole injection layers (HIL-free, GO, rGO, and commercial PEDOT:PSS), as illustrated in Fig. 5(a) and (b). For the HIL-free device, an energy level barrier as high as 0.7 eV exists between the ITO anode and TFB, making hole injection extremely challenging. This leads to a substantially elevated turn-on voltage of 4.0 V and an exceedingly low EQE. After introducing GO as the HIL, it establishes a more matched energy level ladder between ITO and TFB, facilitating hole injection. Consequently, the turn-on voltage decreases to 3.5 V, though the overall performance remains relatively low. When thermally reduced rGO is employed as the HIL, the EQE (11.51%) and CE (12.65 cd A^−1^) of the rGO-based device are significantly superior to those of the GO-based counterpart at the same voltage, and comparable to those of the commercial PEDOT:PSS-based devices fabricated in the same batch, with detailed performance metrics summarized in Table S5. Furthermore, optimization enables the rGO-based QLEDs to achieve optimal EQE and CE of 13.31% and 14.93 cd A^−1^, respectively, fully validating the feasibility of rGO for hole injection and transport (Fig. S9). Besides, thermal imager was used to evaluate the thermal stability of devices with distinct structures. Fig. 5(c) exhibits thermal images of GO-based and rGO-based devices under different operating durations (1, 3, 5, 10 and 20 minutes). The temperature of the emitting region in the GO-based device gradually increased from an initial 21.4 °C to 21.8 °C. In contrast, the emitting region temperature of the rGO-based device remained consistently lower, rising merely to 21.6 °C after 10 minutes and maintaining stability thereafter. The relatively lower operating temperature is attributed to the superior carrier transport properties of rGO, which effectively reduces Joule heating generated by non-radiative recombination, thereby enhancing the thermal stability of the device.^11,39^ The improved thermal stability is of great significance for retarding device aging and prolonging operational lifetime.

## 4. Conclusion

In this study, a high-performance rGO hole injection layer (HIL) was successfully fabricated *via* thermal reduction, and its optimizing effect on QLED performance was systematically explored. Thermal reduction at 160 °C for 30 minutes effectively removed oxygen-containing functional groups from GO, increasing the sp^2^ carbon content from 49.9% to 55.7% and significantly enhancing the electrical conductivity of the resulting rGO. Meanwhile, the work function of rGO was optimized to −5.04 eV, leading to remarkably improved energy level alignment with the ITO anode. Optimized QLED devices with rGO as the hole injection layer exhibit excellent optoelectronic performance: a low turn-on voltage of 2.0 V, a maximum EQE and current efficiency (CE) elevated to 13.31% and 14.93 cd A^−1^, respectively. Further studies revealed that the performance enhancement stems mainly from the substantial improvement in the electrical conductivity of rGO, aided by the optimized energy-level alignment at the ITO/rGO interface. These two factors synergistically facilitate hole injection, reduce carrier injection imbalance, and thereby enhance the overall performance of the devices. Additionally, the rGO-based devices exhibited superior thermal stability, with an operating temperature considerably lower than that of their GO-based counterparts. This study confirms that thermally reduced rGO is an ideal HIL material integrating high performance and stability, offering a viable solution to the hole injection bottleneck in QLED technologies.

## Conflicts of interest

The authors declare that they have no conflict of interest.

## Supplementary Material

RA-016-D6RA00068A-s001

## Data Availability

All data generated or analyzed during the current study are included in this published article and its electronic supplementary information (SI) files. The raw characterization data (*e.g.*, XRD patterns, XPS spectra, FTIR spectra) and key experimental test results supporting the conclusions of this work are fully available within the manuscript and the accompanying SI. No additional restrictions apply to the access of these data. Supplementary information is available. See DOI: https://doi.org/10.1039/d6ra00068a.
