# Supplementary material for: Two-dimensional reduced graphene oxide as high-efficiency hole injection layer for quantum dot light-emitting diodes
Source: RSC Adv. 2026 Mar 12;16(15):13765–73. doi: 10.1039/d6ra00068a (PMC12980520; doi:10.1039/d6ra00068a)
Supplement: RA-016-D6RA00068A-s001 [file RA-016-D6RA00068A-s001.pdf]

Supplementary Information (SI) for RSC Advances.
This journal is © The Royal Society of Chemistry 2026

# Two-Dimensional Reduced Graphene Oxide as High-Efficiency Hole Injection Layer for Quantum Dot Light-Emitting Diodes

Suwen Yang[a], Ning Wang[a] , Yufeng Hu[a] ,Zhidong Lou[a] , Yanbing Hou[a],
Feng Teng*[a], Yu Zhang*[a]

[a]*Key Laboratory of Luminescence and Optical Information, Ministry of Education, School of Physical Science and Engineering, Beijing Jiaotong University, Beijing 100044, China*

**Corresponding author*

*E-mail: fteng@bjtu.edu.cn; zhyu@bjtu.edu.cn*

# Supporting Information

## 1. Experimental Section

1.1 Sample Characterization

The following instruments were employed for systematic characterization in this study:An X-ray Photoelectron Spectrometer (XPS, Escalab 250Xi, Thermo Scientific, USA) was used to analyze the surface chemical states of the materials.A Raman spectrometer (alpha300, WITec, Germany) was utilized to obtain Raman spectra.A field-emission Scanning Electron Microscope (SEM, SU8020, Hitachi, Japan) was employed to observe the sample morphology at an operating voltage of 3.0 kV. An Atomic Force Microscope (AFM, Dimension Icon, Bruker, USA) was used to analyze the surface roughness. Fluorescence spectra were determined with a HORIBA Fluorolog-3 fluorescence spectrometer. Ultraviolet-visible (UV-Vis) absorption spectra were measured using a SHIMADZU UV3600iplus UV-Vis-NIR spectrophotometer. An electrical and optical performance testing system (LQ-50X, Enlitech, Taiwan, China) was adopted to characterize the optoelectronic properties of the QLED devices.

1.2 Synthesis of ZnMgO

Fifteen milliliters of a dimethyl sulfoxide (DMSO) solution containing 0.5 mol/L $Mg(OAc)_2 \cdot 4H_2O$ and $Zn(OAc)_2 \cdot 2H_2O$ (with a mass fraction of $Mg(OAc)_2 \cdot 4H_2O$ of 12.5%) was mixed with 5 milliliters of a 0.55 mol/L tetraethylammonium hydroxide (TEAH) ethanol solution, followed by stirring in ambient air for 1 hour. The resulting product was purified twice using ethanol and n-hexane, and then dispersed in ethanol to form a dispersion with a concentration of 30 mg/mL.

1.3 Experimental Materials and Reagents

Aqueous dispersion of graphene oxide (GO) nanosheets (concentration: 5 mg/mL, flake size: 0.5–5 μm, thickness: 0.8–1.2 nm) was purchased from Nanjing Moke Nano Technology Co., Ltd. Toluene dispersion of red-emitting CdSe/ZnS core-shell quantum dots (QDs) (emission wavelength: 630 nm, concentration: 15 mg/mL) was obtained from Hangzhou Nanjing Tech Co., Ltd. For comparison, the aqueous dispersion of hole injection material poly(3,4-ethylenedioxythiophene):poly(styrenesulfonate) (PEDOT:PSS, Clevios P VP AI 4083) was purchased from Heraeus Group. All solvents employed in the experiment, including acetone, isopropanol, toluene, and anhydrous ethanol, were of analytical grade and purchased from Beijing Marinda Technology Co., Ltd.

## 2.Experimental data

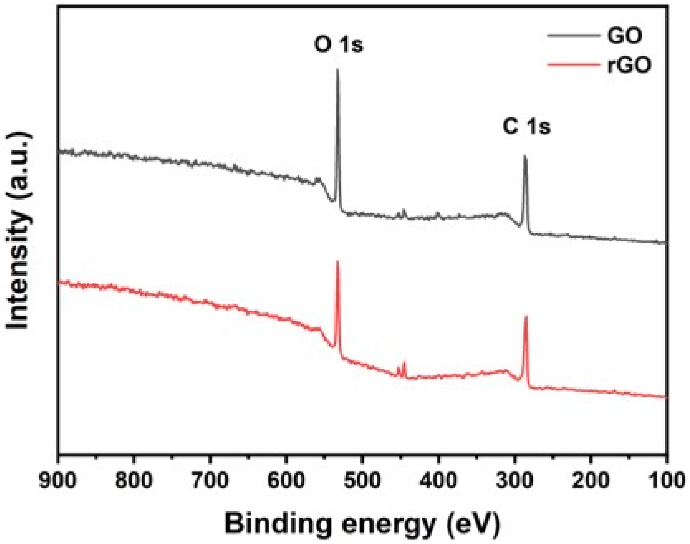

Fig.S1 X-ray photoelectron spectroscopy (XPS) survey spectra of graphene oxide (GO) and reduced graphene oxide (rGO)

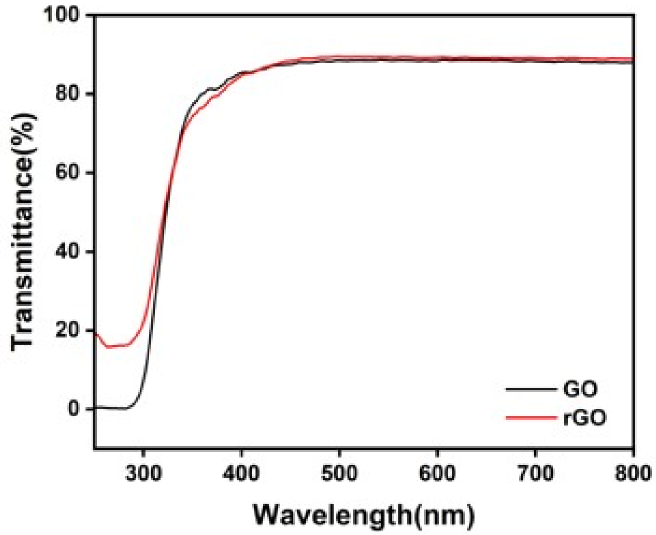


Fig.S2 UV–visible (UV–Vis) transmittance spectra of graphene oxide (GO, black curve) and reduced graphene oxide (rGO, red curve) over a wavelength range of 300–800 nm, which illustrates the impact of the reduction process on the optical transparency of the two materials.

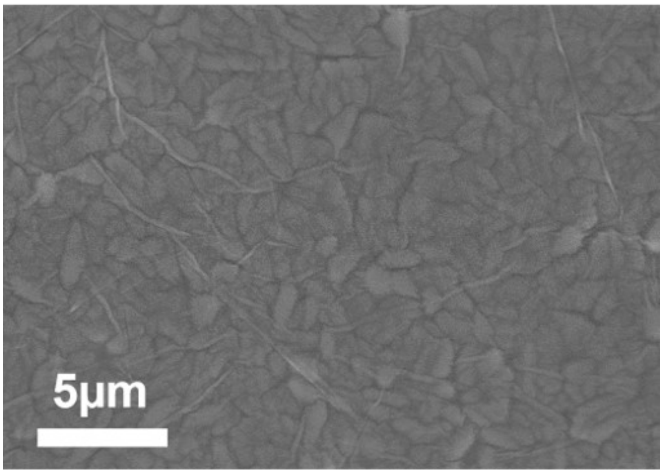


Fig.S3 SEM image of rGO deposited on $SiO_2$/Si.

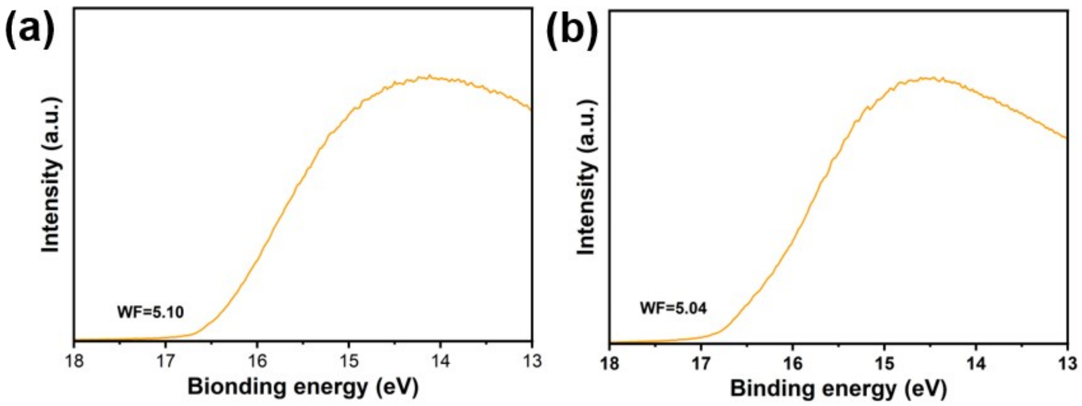

Fig.S4 Ultraviolet photoelectron spectroscopy (UPS) analysis of work function. (a) UPS spectrum and work function (WF=5.10 eV) of GO;(b) UPS spectrum and work function (WF=5.04 eV) of reduced graphene oxide (rGO).

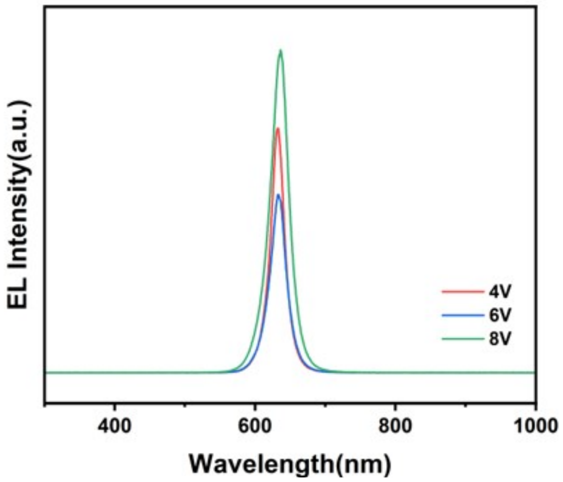


Fig.S5 Electroluminescence (EL) spectra of quantum dot light-emitting diodes (QLEDs) measured at varying driving voltages.

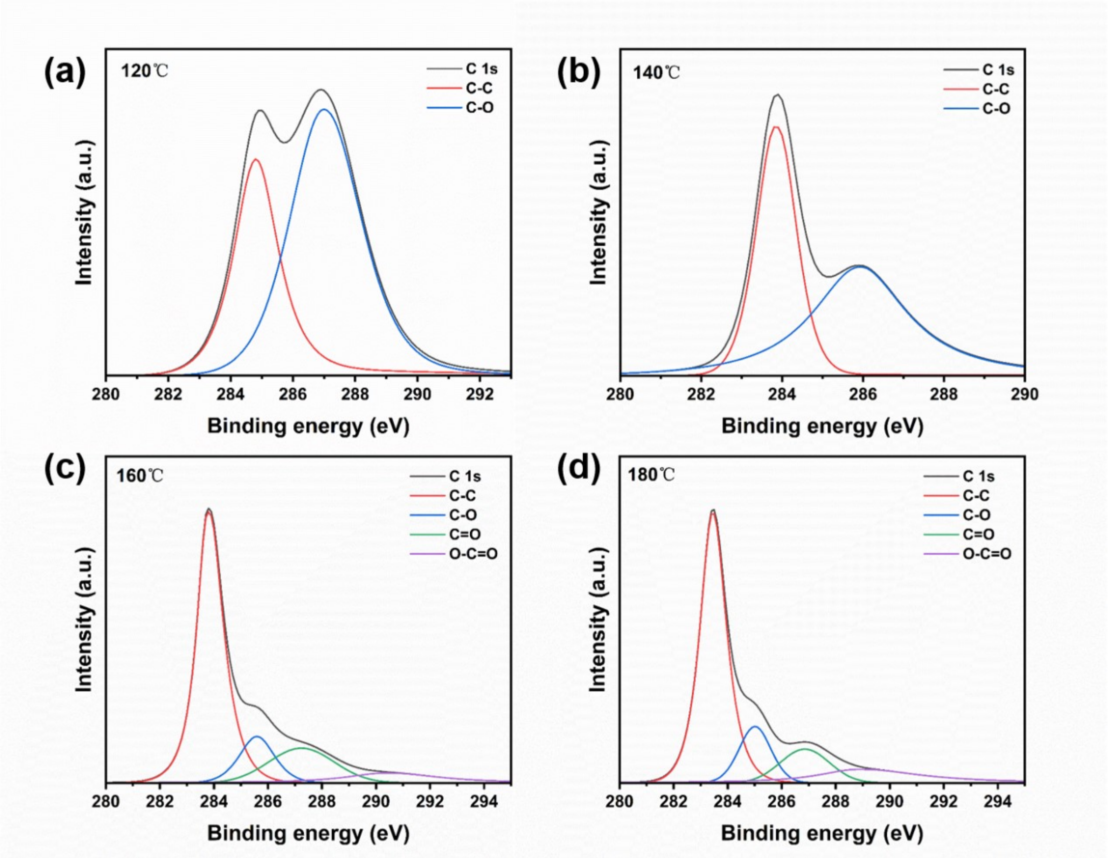


Fig.S6 High-resolution C 1s XPS spectra of rGO films annealed at 120–180 °C for 30 min: (a) 120 °C, (b) 140 °C, (c) 160 °C, (d) 180 °C.

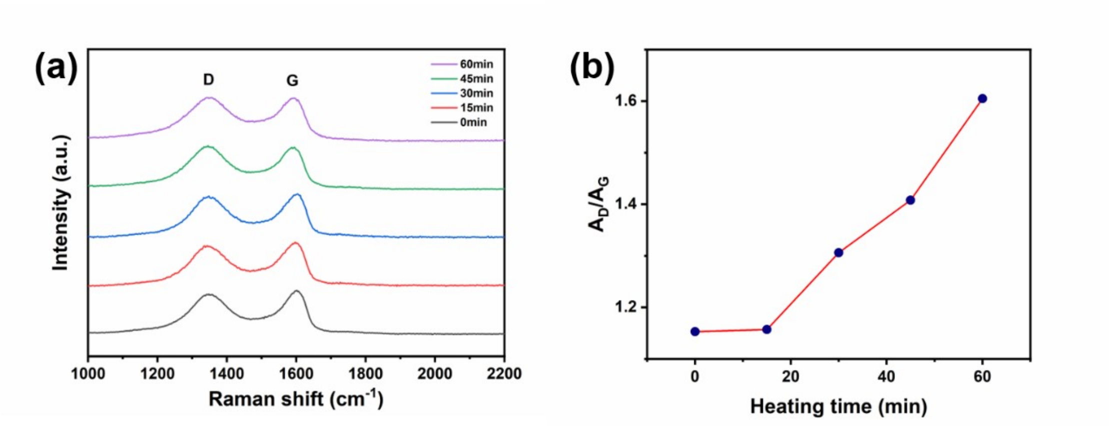


Fig.S7 Raman spectroscopy analysis of reduced graphene oxide (rGO). (a) Raman

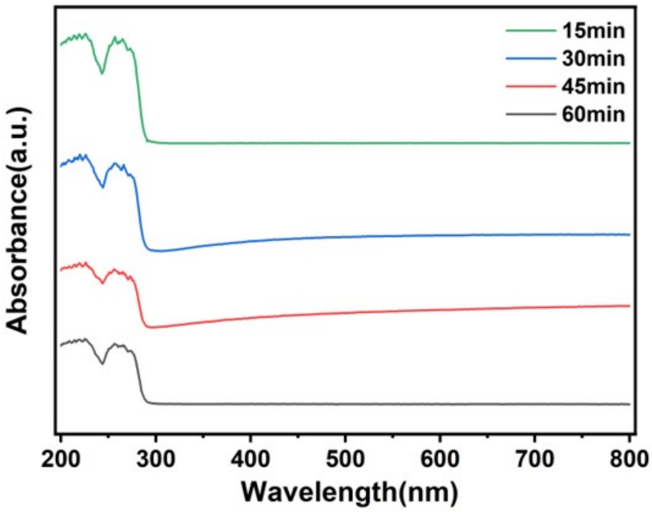


spectra of rGO at different heating reduction times (0 min, 15 min, 30 min, 45 min, 60 min), showing the peak area changes of D peak (~1350 cm$^{-1}$) and G peak (~1580 cm$^{-1}$) for characterizing the defect and graphitization degree of the material. (b) Plot of the Raman D-to-G peak area ratio ($A_D/A_G$) as a function of thermal reduction time, illustrating the evolution of defect states during the rGO reduction process.

Fig.S8 UV-Vis absorption spectra of reduced graphene oxide under different thermal annealing times (15min, 30min, 45min, 60min)

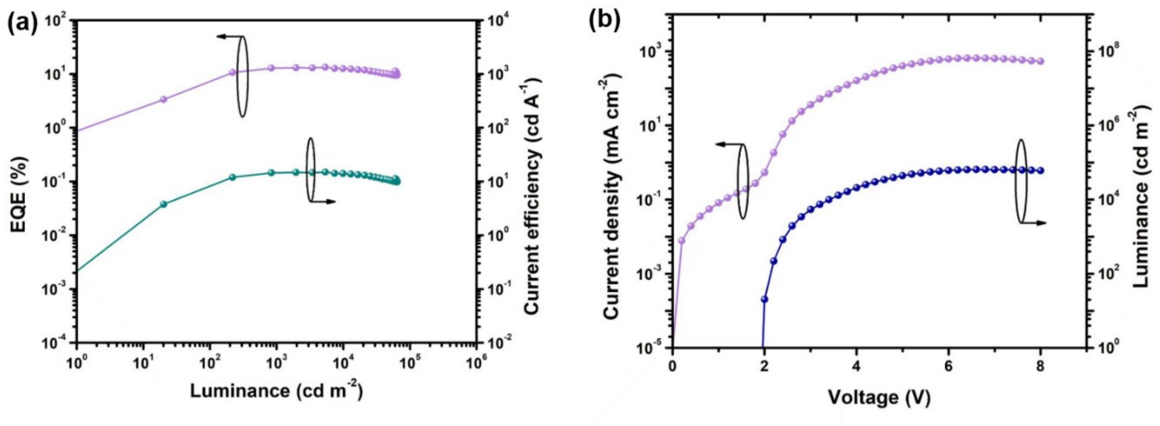


Fig.S9 (a) Current density and luminance versus voltage (J-V-L) curves of rGO-based device; (b) External quantum efficiency (EQE) and Current efficiency (CE) versus luminance plots of rGO-based device.

Table S1. Fourier Transform Infrared Spectroscopy (FTIR) Characteristic Peak Data.

| NO. | Wavenumber | Function Group |
|---|---|---|
| 1 | 1005 | -C-O- |
| 2 | 1192 | -C-O-C |
| 3 | 1440 | -O-H- |
| 4 | 1634,1737 | C=C- |
| 5 | 2852,2927 | $-CH_2-$ |

Table S2. Electrical and optical performance parameters of rGO-based QLEDs with different annealing temperatures.

| Annealing temperature(°C) | $V_{on}$（V） | Current density(mA cm$^{-2}$) (6V) | $L_{max}$（cd m$^{-2}$） | EQE $_{max}$(%) | $CE_{max}$（%） |
|---|---|---|---|---|---|

| | | | | | |
|---|---|---|---|---|---|
| 120 | 2.6 | 14.38 | 1168 | 9.21 | 10.12 |
| 140 | 2.2 | 78.21 | 6395 | 9.94 | 11.06 |
| 160 | 2.0 | 533.31 | 47013 | 11.74 | 13.30 |
| 180 | 2.0 | 1411.99 | 81975 | 7.76 | 8.61 |

Table S3. Raman characteristic peaks and peak area ratios of GO and rGO under different annealing times.

Table S4. Electrical and Optical Performance Parameters of rGO-based QLEDs with different Annealing Times.

| **Annealing time(min)** | **$V_{on}$（V）** | **Current density(mA cm$^{-2}$) (8V)** | **$L_{max}$（cd m$^{-2}$）** | **EQE $_{max}$(%)** | **$CE_{max}$（%）** |
|---|---|---|---|---|---|
| 0 | 2.6 | 108.84 | 3296 | 4.95 | 5.46 |
| 15 | 2.0 | 812.56 | 44755 | 8.79 | 9.35 |
| 30 | 2.0 | 1189.18 | 81697 | 11.46 | 11.63 |
| 45 | 2.2 | 1458.42 | 76689 | 8.45 | 9.04 |
| 60 | 2.4 | 2034.08 | 52545 | 5.75 | 6.22 |

| **Heating time** | **D Peak** | **G Peak** | **$A_D/A_G$** |
|---|---|---|---|
| 0 | 1350 | 1601 | 1.153 |
| 15 | 1343 | 1597 | 1.157 |
| 30 | 1348 | 1604 | 1.306 |
| 45 | 1345 | 1594 | 1.408 |
| 60 | 1343 | 1592 | 1.605 |

Table S5. Summary of device performance with different HILs.

| **HILs** | **Von(V)** | **$L_{max}$(cd m$^{-2}$)** | **$EQE_{max}$ (%)** | **$CE_{max}$(cd/A)** |
|---|---|---|---|---|
| None | 4.2 | 22666 | 5.59 | 5.95 |
| GO | 2.8 | 7157 | 8.07 | 8.99 |
| **rGO** | **2.0** | **120517** | **11.51** | **12.65** |
| PEDOT:PSS | 2.0 | 196813 | 12.80 | 14.13 |
